# Large-scale evaluation of soybean germplasm reveals geographic patterns in shade tolerance and identifies elite genotypes for intercropping systems

**DOI:** 10.1186/s12870-025-07121-5

**Published:** 2025-08-18

**Authors:** Fengyi Zhang, Huilong Hong, Xiulin Liu, Xueyang Wang, Chunlei Zhang, Kezhen Zhao, Rongqiang Yuan, Ahmed M. Abdelghany, Bixian Zhang, Sobhi F. Lamlom, Honglei Ren

**Affiliations:** 1 Soybean Research Institute of Heilongjiang Academy of Agriculture Sciences, Harbin, 150086 China; 2 National Key Facility for Crop Gene Resources and Genetic Improvement, Institute of Crop Sciences, Chinese Academy of Agricultural Sciences, Beijing, 100081 China; 3 Crop Science Department, Faculty of Agriculture, Damanhour University, Damanhour, 22516 Egypt; 4 Institute of Biotechnology of Heilongjiang Academy of Agricultural Sciences, Harbin, 150023 China; 5 Plant Production Department, Faculty of Agriculture Saba Basha, Alexandria University, Alexandria, 21531 Egypt

**Keywords:** Soybean, Shade tolerance, Intercropping, Tolerance index, Machine learning

## Abstract

Shade tolerance is crucial for developing soybean varieties that are well-suited for intercropping systems. This study evaluated 460 soybean accessions from a global germplasm collection at two contrasting locations in China: Heilongjiang (HLJ) and Inner Mongolia (NM). The experiment used a split-plot design within a randomized complete block setup, with three replications at each site. The main plots consisted of two treatments (control and shade), and the 460 soybean accessions were randomly assigned as subplots within each treatment. Soybean genotypes were evaluated under shaded conditions (maize-soybean intercropping) and unshaded conditions (soybean monoculture) across multiple locations to assess shade tolerance and agronomic performance. Six agronomic traits were measured: plant height (PH), basal pod height (BPH), number of nodes per plant (NNP), branch number (BN), pod number per plant (PNP), and seed yield per plant (SYP). Results showed highly significant effects of location and treatment (*p* < 0.001) on all traits, with complex interactions between location and treatment for reproductive traits. Shade treatment triggered typical avoidance responses, increasing PH by 7.5–11.7% and BPH by 11.5–37.5%, while significantly reducing reproductive output, with PNP decreasing by 8.2–28.9% and SYP dropping by 23.9% at HLJ. Path analysis revealed a fundamental reorganization of trait contributions under shade, with NNP becoming the most critical factor for yield. Geographic differences were notable, with NM accessions showing higher shade tolerance indices compared to HLJ populations. The study identified 120 elite, stable cultivars (13.7%) that maintained consistent high performance and 100 shade-adaptive cultivars (11.4%) demonstrating enhanced plasticity. These findings provide insights into the genetic architecture of soybean shade tolerance and offer a framework for evaluating germplasm. The identified genotypes are valuable resources for developing intercropping-compatible varieties and promoting sustainable agriculture.

**Supplementary Information:**

The online version contains supplementary material available at 10.1186/s12870-025-07121-5.

## Introduction

Soybean (*Glycine max* L. Merr.) represents one of the world’s most economically significant leguminous crops, providing essential protein and oil resources that sustain global food security and industrial applications [1–3]. As the fourth-largest crop by production area globally, soybeans contribute approximately 60% of the world’s protein meal and 30% of vegetable oil consumption, making their agricultural optimization a critical priority for meeting the nutritional demands of an expanding global population [4]. However, contemporary agricultural systems face unprecedented challenges from climate variability, land use intensification, and the urgent need to develop sustainable cropping practices that can maintain productivity under increasingly constrained environmental conditions [1, 4].

Light availability emerges as a fundamental environmental factor governing photosynthetic efficiency, morphological development, and ultimately yield performance in soybean cultivation systems [5, 6]. Optimal light interception and utilization are crucial for maximizing photosynthetic carbon assimilation, which directly influences biomass accumulation, reproductive development, and grain filling processes [7]. In natural and agricultural ecosystems, soybeans frequently encounter suboptimal light conditions due to various factors including intercropping systems [8–10], agroforestry practices [11], and dense canopy structures [12, 13]. These shade-stressed environments trigger complex physiological responses that fundamentally alter plant architecture, resource allocation patterns, and competitive interactions, often resulting in significant yield penalties that threaten agricultural sustainability and economic viability [14, 15].

The physiological mechanisms underlying shade stress responses in soybeans involve intricate regulatory networks that coordinate morphological plasticity with metabolic adjustments to optimize survival under light-limited conditions [16]. When exposed to reduced light intensity, soybeans typically exhibit characteristic shade avoidance responses including stem elongation, reduced branching, altered leaf morphology, and modified reproductive timing [17–21]. These morphological adaptations represent evolutionary strategies to maximize light capture through increased height and enhanced light interception efficiency, yet they often compromise structural stability and resource allocation to reproductive organs (Kim et al., 2022; Crop Science, 2021). Understanding these adaptive mechanisms is essential for developing cultivars that can maintain productivity under shade stress while preserving desirable agronomic characteristics such as lodging resistance and harvest index optimization [22].

Evaluating agronomic traits under controlled shade stress conditions is pivotal for unraveling the complex physiological and developmental responses that differentiate shade-tolerant soybean cultivars from sensitive ones. Plant height, for instance, often increases as part of the shade avoidance syndrome, where stem elongation is stimulated to outgrow competing vegetation and optimize light capture [23, 24]. However, this elongation can come at the expense of mechanical strength, increasing susceptibility to lodging, which negatively impacts yield stability. Branching patterns are equally critical; reduced branching under shade conditions reflects a strategic reallocation of resources away from lateral growth towards vertical extension [25, 26]. This shift alters canopy architecture and influences light interception efficiency, but may limit the number of reproductive sites, thereby affecting yield potential. Regarding node development, which determines the number of potential flowering and pod-setting sites, is also sensitive to light availability. Shade stress can delay node formation or reduce node number, constraining the plant’s reproductive capacity [21]. This effect underscores the importance of understanding developmental timing and its plasticity in response to light stress. Reproductive organ formation, including flower initiation and pod development, is often compromised under shade due to altered hormonal signaling and resource limitations [27]. These changes can lead to reduced pod set and seed filling, directly impacting grain yield and quality. By integrating these morphological and physiological traits, researchers can better characterize the shade tolerance spectrum across soybean germplasm. This comprehensive phenotyping, especially when conducted across diverse environmental conditions, could confer resilience to low-light environments. Ultimately, agronomic trait evaluation under shade stress not only advances our fundamental understanding of plant adaptation but also supports sustainable agricultural practices by enabling the selection of soybean varieties optimized for performance under suboptimal light conditions.

Genetic diversity in soybean collections provides essential resources for studying shade tolerance and developing cultivars suitable for light-limited environments. This research evaluated 460 soybean cultivars under shade and monoculture conditions in China in 2022 at two locations: Heilongjiang and Inner Mongolia. Shade was created through intercropping maize and soybean, with six agronomic traits measured: plant height, basal pod height, node number, branch number, pod number, and yield. The aim was to understand how soybeans respond morphologically and reproductively to shade, identify tolerant cultivars, and analyze genotype-by-environment interactions, offering valuable insights for improving soybean adaptation to low-light conditions.

## Materials and methods

### Plant materials, experimental design, and statistical analysis

**Fig. 1:**
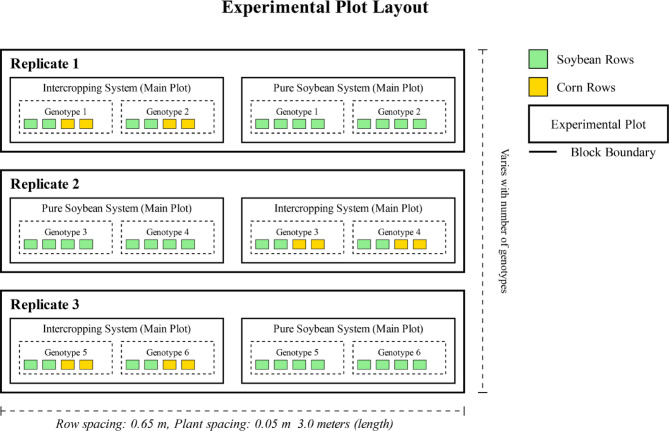
Experimental plot layout for comparison of soybean genotypes under intercropping and pure soybean cropping systems using a split plot design with three replicates

A collection of 460 soybean genotypes was obtained from the germplasm bank at the Chinese Academy of Agricultural Sciences (Table S1). These genetic resources represent a global sampling from 8 ecoregions across North America, Europe, and Asia. The collection is geographically stratified, with China providing the majority (314 accessions), followed by Russia (46), USA (33), Central Europe (37), Canada (19), South Korea (6), and Japan (5). Detailed characterization data for all accessions are included in Table S1. The complete germplasm panel is stored at the Chinese National Soybean Gene Bank (CNSGB). Field experiments were conducted during the 2022 growing season at two ecologically contrasting sites to capture diverse environmental conditions: Minzhu Town (Daowai District, Harbin City, Heilongjiang Province), representing the northern temperate soybean production region, and Ulanhot City (Xing’an League, Inner Mongolia), representing the semi-arid transitional zone. At each site, the experiment used a split-plot design within a randomized complete block design (RCBD) with three replications to ensure statistical validity. The main plots were assigned to cropping system treatments: (i) an intercropping system with a maize-soybean relay involving alternating strips of two maize rows and four soybean rows, and (ii) a monoculture control reflecting traditional soybean farming. Within each main plot, subplots were designated for different soybean genotypes, with each pair of neighboring soybean rows forming a single genotypic unit (Fig. 1). The maize cultivar Sudan 128’ was used in the intercropping system due to its high plant height, appropriate canopy architecture for creating shade conditions, and widespread adoption in the study regions, ensuring practical relevance for local farming systems.

Soybean accessions were randomly assigned within each subplot to minimize positional effects and environmental variation. The intercropping setup was designed to induce controlled shade stress through light competition from the taller maize crop, mimicking realistic relay cropping conditions. Statistical analyses were conducted independently for each location and treatment system to account for site-specific environmental variations and maintain the integrity of the replicated experimental structure across contrasting agroecological zones. The following linear model was used to analyze each trait:

$$\:{\varvec{Y}}_{\varvec{i}\varvec{j}\varvec{k}}=\varvec{\mu\:}+{\varvec{R}}_{\varvec{i}}+{\varvec{C}}_{\varvec{i}}+{\varvec{\delta\:}}_{\varvec{i}\varvec{j}}+\varvec{G}\varvec{k}+\left(\varvec{C}\varvec{G}\right)\varvec{j}\varvec{k}\:+{\varvec{\epsilon\:}}_{\varvec{i}\varvec{j}\varvec{k}}$$

where: Y_ijk_ is the observed phenotypic value for the k_th_ genotype in the j_th_ cropping system within the i_th_ replication, µ is the overall mean, R_i_ is the random effect of the i_th_ replication, C_j_ is the fixed effect of the j_th_ cropping system (intercropping vs. monoculture), δ_ij_ is the random main plot error (whole plot error), G_k_ is the fixed effect of the k_th_ genotype, (CG)_jk_ is the interaction effect between cropping system and genotype, ε_ijk_ is the random subplot error.

Soybean plants were spaced at 15 cm within rows and 40 cm between rows. Fertilizer application included basal fertilization (45 kg N ha¹, 75 kg P₂O₅ ha¹, 60 kg K₂O ha¹) and topdressing (30 kg N ha¹) at the flowering stage. Field management practices included regular weeding, pest control implemented according to integrated pest management principles, and irrigation as needed to maintain optimal soil moisture levels. Table S2 providing detailed climatic data (mean temperature, precipitation, and solar radiation).

### Phenotypic evaluation

To quantify the physiological and morphological responses to shade stress, six key agronomic traits were systematically evaluated: plant height (PH), basal pod height (BPH), node number per plant (NNP), branch number (BN), pod number per plant (PNP), and seed yield per plant (SYP). Phenotypic evaluations were conducted after plants reached physiological maturity, utilizing 10 randomly selected plants from each genotype within each plot to ensure representative sampling. For PH, readings were taken as the vertical distance from the soil surface to the uppermost growing point of the main stem, using a standardized measuring tape. Individual measurements were averaged across the 10 plants per genotype sampled. For BPH, the critical harvest height parameter for mechanical harvesting efficiency was determined by measuring the distance from the soil surface to the insertion point of the lowest pod-bearing node. Regarding the determination of NNP, it was quantified by systematically counting all nodes along the main stem from the cotyledonary node to the terminal growing point, providing an indicator of plant developmental architecture. Also, BN was assessed by enumerating all primary lateral branches originating from the main stem nodes, excluding secondary branching structures. A comprehensive counting of all pods, including both fully mature and developing structures, across each sampled plant was evaluated to capture total PNP as a reproductive output. Finally, SYP was determined by harvesting, cleaning, and weighing all seeds from individual plants using precision electronic scales, with moisture content standardized to facilitate accurate yield comparisons. All trait measurements were replicated across the 10 plants per plot and subsequently averaged to generate robust genotypic means, with data collection protocols maintained consistently across both experimental locations and treatment systems.

### Data analysis

Statistical analyses were performed using R software (version 4.3.0) within the RStudio integrated development environment to ensure reproducible and robust analytical workflows. For each of the six agronomic traits (PH, BPH, NNP, BN, PNP, and SYP), phenotypic data were subjected to a comprehensive statistical evaluation following a structured analytical framework. Analysis of variance (ANOVA) was conducted separately for each experimental location using the RCBD model. Prior to conducting the ANOVA, the data normality and homogeneity of variance assumptions were verified using the Shapiro-Wilk and Levene’s tests, respectively. To quantify shade stress responses across the germplasm collection, the tolerance index was calculated for each trait using the formula: Tolerance Index (TI) = (Trait value under shade stress/Trait value under control conditions). This standardized metric is expressed as a decimal proportion (not percentage), with values closer to 1 indicating greater shade tolerance. Values equal to 1.0 indicate no performance change, values below 1.0 indicate performance reduction, and values above 1.0 indicate enhanced performance under shade conditions. This approach enabled direct comparison of shade tolerance across different genotypes and traits within the collection. All statistical computations, data manipulation, and visualization procedures were executed within the R statistical computing environment to maintain analytical transparency and reproducibility.

## Results

### Patterns of location, shade treatments, and their interaction effects

**Fig. 2:**
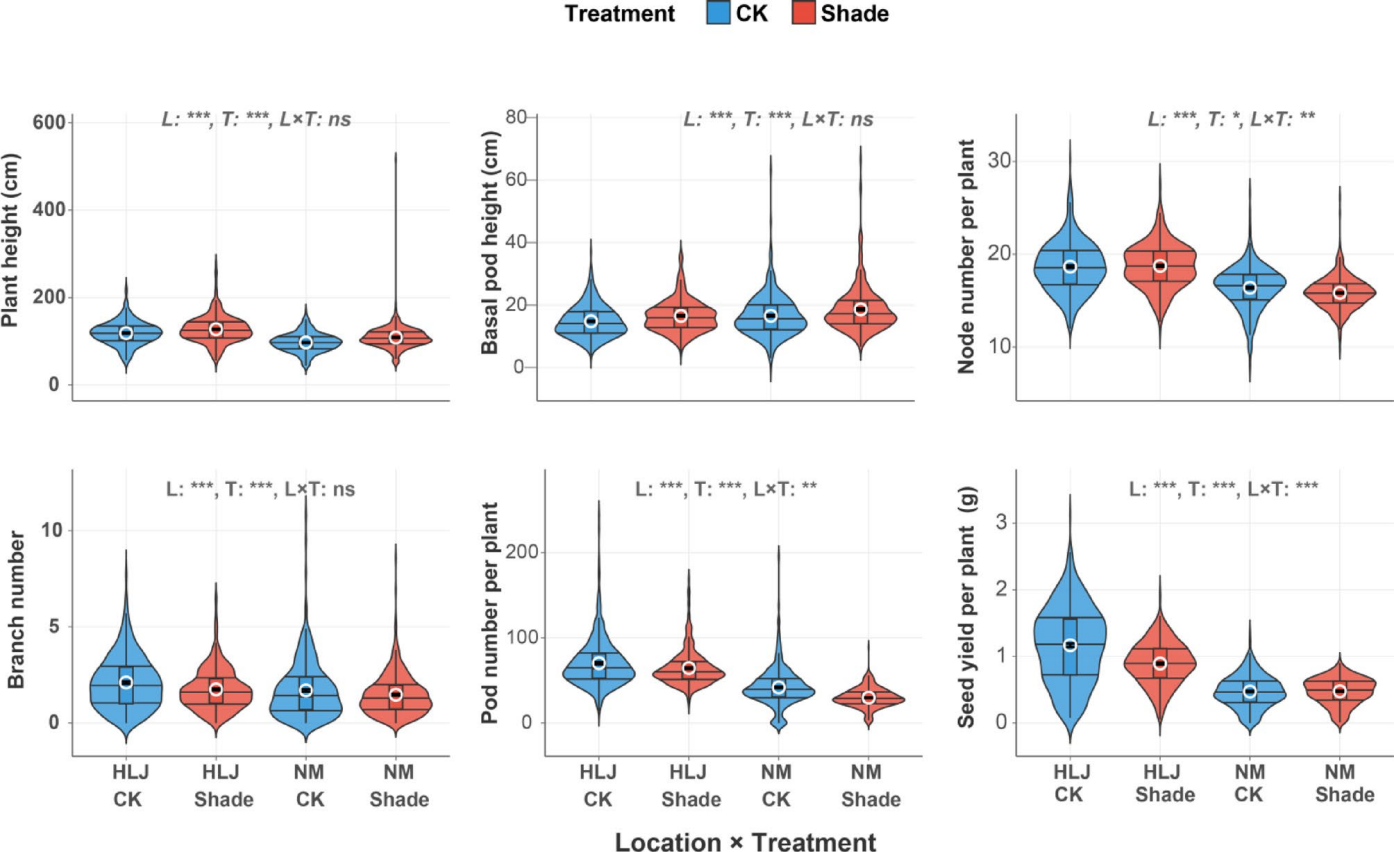
Location × treatment interaction effects on phenotypic traits in soybean grown under control and shade conditions at two geographical locations in China. Violin plots display the distribution, median values (black dots), and quartiles for six traits across four treatment combinations: Heilongjiang control (HLJ_CK), Heilongjiang shade (HLJ_Shade), Inner Mongolia control (NM_CK), and Inner Mongolia shade (NM_Shade). Blue violins represent the Heilongjiang location, and red violins represent the Inner Mongolia location. Statistical significance levels are indicated as: *** *p* < 0.001, ** *p* < 0.01, * *p* < 0.05, ns = non-significant for location (L), treatment (T), and location × treatment (L × T) interaction effects. Violin width represents the density distribution of data points, with wider sections indicating a higher frequency of observations at those values

The two-way ANOVA revealed significant main effects and complex interaction patterns across the six measured traits in the 460 soybean accessions evaluated under control and shade treatments at HLJ and NM locations. Highly significant location effects (*p* < 0.001) were observed for all six traits, with substantial differences between HLJ and NM sites (Fig. 2). Under control conditions, HLJ showed superior performance with higher PH (118.60 vs. 97.17 cm), BPH (14.80 vs. 13.53 cm), NNP (18.66 vs. 16.39), BN (2.11 vs. 1.70), PNP (70.17 vs. 41.81), and SYP (1.17 vs. 0.47) compared to NM. These differences reflect the distinct environmental conditions between the northern locations, including variations in temperature regimes, photoperiod, and soil characteristics.

Similarly, treatment exhibited highly significant effects (*p* < 0.001) on PH, BPH, NNP, BN, PNP, and SYP, indicating that shading conditions substantially influence plant morphology and reproductive output compared to control conditions. The shade treatment resulted in contrasting responses between locations (Fig. 2). At HLJ, shade treatment increased PH from 118.60 to 127.49 cm (+ 7.5%) and BPH from 14.80 to 16.50 cm (+ 11.5%), while reducing BN from 2.11 to 1.75 (−17.1%), PNP from 70.17 to 64.40 (−8.2%), and SYP from 1.17 to 0.89 (−23.9%). For NNP, it remained relatively stable (18.66 to 18.75). At NM, shade treatment similarly increased pH from 7.17 to 8.55 (+ 11.7%) and BPH from 13.53 to 18.60 cm (+ 37.5%), while dramatically reducing PNP from 41.81 to 29.72 (−28.9%) and BN from 1.70 to 1.47 (−13.5%). NNP decreased from 16.39 to 15.84 (−3.4%), and SYP remained relatively stable (0.47 to 0.48). Noteworthy, the analysis revealed significant location × treatment interactions for three traits: NNP (*p* < 0.01), PNP (*p* = 0.01), and SYP (*p* < 0.001). Conversely, PH, BPH, and BN showed non-significant interactions (*p* > 0.05), suggesting that the treatment effects on these morphological parameters were consistent across both locations.

### Comparative response of soybean traits to shade across two locations

**Fig. 3:**
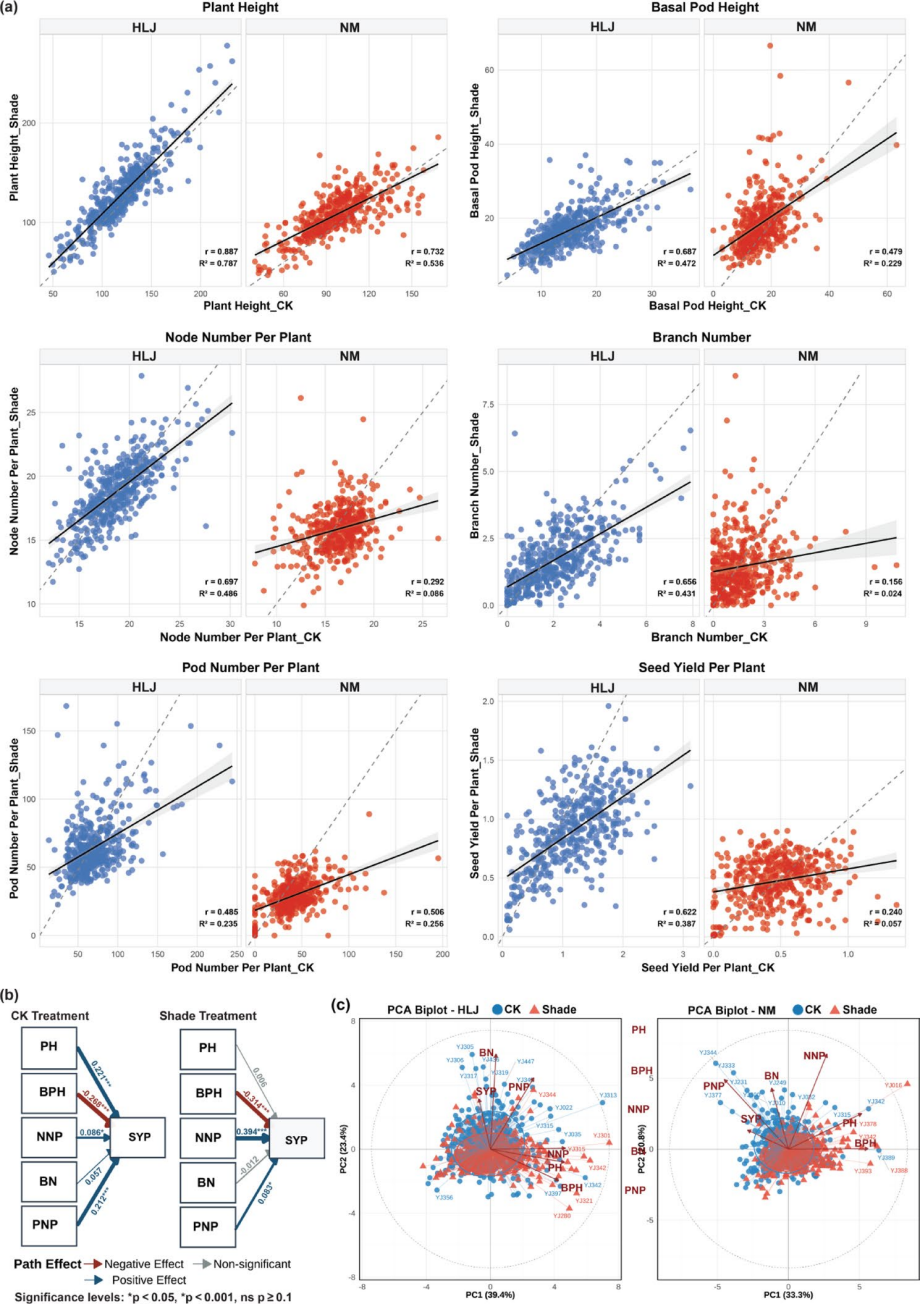
Comparative analysis of soybean phenotypic traits under control (CK) and shade treatments across two locations (HLJ and NM). (**a**) Scatterplots depicting the correlations between six soybean traits under CK and shade conditions, faceted by location. Traits include plant height (PH), basal pod height (BPH), node number per plant (NNP), branch number (BN), pod number per plant (PNP), and seed yield per plant (SYP). Solid black lines represent linear regressions, and dashed gray lines denote 1:1 reference lines. Correlation coefficients (r), coefficients of determination (R²). (**b**) Path coefficient analysis for both CK (left) and shade (right) treatments showing direct and indirect effects of five morphological traits (PH, BPH, NNP, BN, PNP) on seed yield per plant (SYP). Arrows indicate direction and magnitude of effects: blue arrows represent positive effects, red arrows represent negative effects, and gray arrows represent non-significant effects. Significance levels: ***p < 0.001, **p < 0.01, *p < 0.05. (**c**) Principal component analysis (PCA) biplots of soybean cultivars under CK (blue circles) and shade (red triangles) treatments for HLJ (left) and NM (right) locations. Vectors represent trait loadings on the first two principal components, with longer vectors indicating stronger contributions. The spatial distribution of cultivars reflects multivariate phenotypic variation and treatment responses across locations

To further evaluate genotype-by-environment interactions and phenotypic plasticity under CK and shade treatments, pairwise trait comparisons between CK and shade treatments within the same genotype revealed significant positive correlations (*p* < 0.001) for all six traits across both environments (Fig. 3a). However, trait-specific responses to shade stress varied significantly between locations, with correlation patterns between control and shade conditions differing markedly by location, revealing distinct environmental influences on trait expression patterns and confirming substantial genotype × environment interactions. The results showed that PH exhibited the strongest correlations in both locations (HLJ: *r* = 0.887, R² = 0.787; NM: *r* = 0.732, R² = 0.536), with HLJ showing superior correlation strength. Also, SYP demonstrated strong correlation in HLJ (*r* = 0.622, R² = 0.387) but weak correlation in NM (*r* = 0.240, R² = 0.057). Architectural traits showed pronounced location-specific responses, demonstrating that some traits showed consistent responses across locations while others were highly location-specific. For example, BPH correlations were moderate in HLJ (*r* = 0.687, R² = 0.472) but weak in NM (*r* = 0.479, R² = 0.229), while node number per plant exhibited strong correlation in HLJ (*r* = 0.697, R² = 0.486) versus weak correlation in NM (*r* = 0.292, R² = 0.086). Branch number showed the most dramatic location difference, with moderate correlation in HLJ (*r* = 0.656, R² = 0.431) but negligible correlation in NM (*r* = 0.156, R² = 0.024). Interestingly, PNP was the only trait showing relatively similar correlation strengths between locations (HLJ: *r* = 0.485, R² = 0.235; NM: *r* = 0.506, R² = 0.256).

To quantify the direct contributions of architectural traits to final seed yield, path analysis was conducted under both CK and shade treatments (Fig. 3b). Under CK conditions, PH had the strongest positive direct effect on SYP (path coefficient = 0.221, *p* < 0.001), followed by PNP (0.212, *p* < 0.001) and NNP (0.086, *p* < 0.05). Interestingly, BPH showed a significant negative effect on yield (−0.268, *p* < 0.001), suggesting that cultivars with lower BPH had higher yield efficiency. Notably, BN had a weak positive but non-significant effect (0.057). Under shade treatment, trait contributions to yield showed a notable shift. For example, NNP (0.394, *p* < 0.001) emerged as the most influential trait, implying that the capacity to produce more nodes plays a critical role in reproductive resilience under reduced light availability. In contrast, the direct effect of PH became non-significant (0.006), reflecting diminished benefit from elongation growth in shaded environments. The negative influence of BPH intensified (−0.314, *p* < 0.001), while the positive effect of PNP decreased (0.083). These shifts between the two-contrasting light conditions emphasize the altered physiological priorities in soybeans exposed to light stress and highlight the trait-specific contributions to yield stability across light environments (Fig. 3b).

To visualize multivariate trait variation and treatment effects across the two study locations, principal component analysis (PCA) was employed (Fig. 3c). In HLJ (Fig. 3c, left**)**, the first two PCs accounted for 62.8% of the total phenotypic variance (PC1: 39.4%, PC2: 23.4%). The biplot revealed moderate separation between CK and shade treatments, with CK genotypes occupying a broader range across the plot. Trait vectors indicated that SYP, PNP, and BN were more aligned with PC2, whereas PH, NNP, and BPH loaded more strongly on PC1. This suggests that architectural traits were the major contributors to genotypic variation in HLJ, while reproductive traits also played a notable role. In NM (Fig. 3c, right), PC1 and PC2 explained 54.1% of the variance (PC1: 33.3%, PC2: 20.8%). Here, a more distinct clustering of shade-treated genotypes was observed on the right side of the plot, indicating a clearer treatment effect. Architectural traits such as NNP and BPH had stronger associations with PC1 compared to HLJ, while SYP and PNP continued to align closely and contribute positively to variance along PC2, suggesting greater plasticity and environment-specific trait shifts in NM, and underscoring the importance of local adaptation and trait integration in determining soybean performance under light-limited conditions (Fig. 3c).

### Assessment of shade tolerance variability and population structure in soybean germplasm

**Fig. 4:**
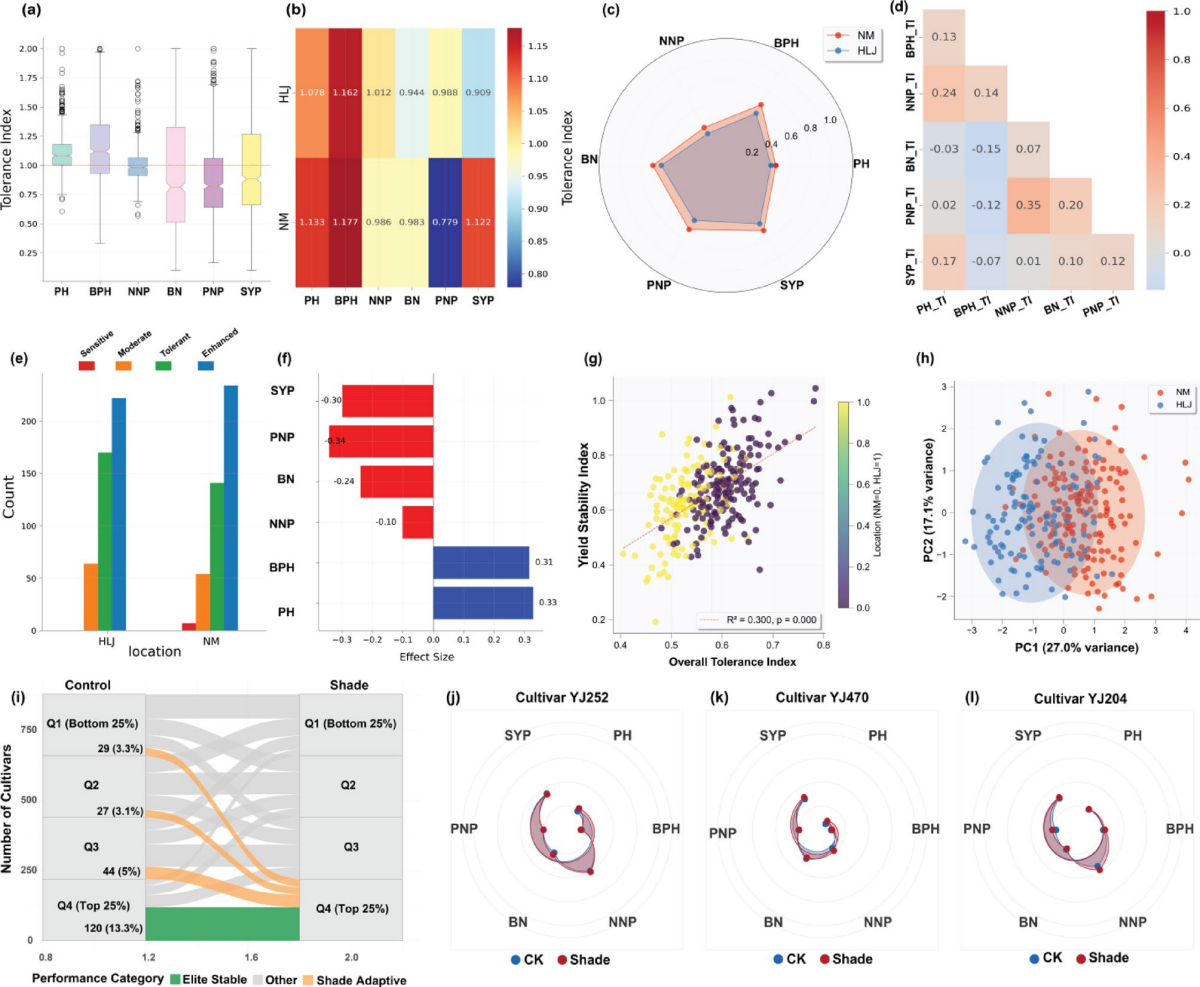
Comprehensive shade tolerance profiling of 460 soybean accessions across two geographic locations. (a) Classification of accessions into four shade tolerance categories—highly-sensitive (red), sensitive (orange), moderate (green), and tolerant (blue)—in Heilongjiang (HLJ) and Inner Mongolia (NM). (b) Effect sizes (Cohen’s d) of shade impact on six agronomic traits. (c) Correlation between overall tolerance index and yield stability index, colored by geographic origin. (d) Radar chart showing average trait-specific tolerance indices for HLJ and NM populations. (e) Violin plots of tolerance index distributions across traits. (f) Principal Component Analysis (PCA) showing population structure and geographic differentiation. (g) Boxplots of trait-specific tolerance indices by location. (h) Correlation matrix of trait tolerance indices. (i) Sankey diagram illustrating the classification of cultivars into elite stable and shade-adaptive groups. Elite stable cultivars (*n* = 120, 13.7%) consistently ranked in Q4 under both conditions, while shade-adaptive cultivars (*n* = 100, 11.4%) showed substantial improvement from lower quartiles in control to Q4 under shade. (j–l) Polar plots depicting shade response profiles of three representative cultivars (YJ252, YJ470, YJ204) under control (blue) and shade (red) conditions

To comprehensively evaluate shade tolerance across the soybean germplasm collection, we analyzed tolerance index for the entire collection of soybean accession as shown in **Table ****S3**. Then, we performed the tolerance index distributions, geographic patterns, and phenotypic responses for six key agronomic traits under shade conditions. The probability density distributions for each trait tolerance index were analyzed and presented in Fig. 4a. PH tolerance showed a relatively narrow distribution (mean: 1.105 ± 0.162, range: 0.605-2.000) centered around 1.0-1.2, indicating consistent shade avoidance responses across genotypes. BPH tolerance exhibited the broadest distribution (mean: 1.169 ± 0.339, range: 0.332-2.000) with high variability and numerous outliers, suggesting substantial genetic variation in vertical positioning responses. NNP tolerance displayed a moderate distribution (mean: 0.999 ± 0.143, range: 0.569-2.000) centered around 1.0, while BN tolerance showed wide variation (mean: 0.963 ± 0.575, range: 0.100-2.000) with extensive outliers extending from 0.1 to 2.0. PNP tolerance demonstrated moderate variation (mean: 0.889 ± 0.357, range: 0.169-2.000), and SYP tolerance exhibited a broad distribution (mean: 1.014 ± 0.497, range: 0.100-2.000) with strong genetic diversity across the population.

The heatmap analysis provided comprehensive insights into tolerance index distributions by location and trait (Fig. 4b). PH showed higher tolerance in NM (1.133 ± 0.184) compared to HLJ (1.078 ± 0.132), while BPH revealed similar patterns with NM (1.177 ± 0.385) outperforming HLJ (1.162 ± 0.290). NNP and BN displayed nearly identical tolerance indices between locations (HLJ: 1.012 ± 0.113 vs. NM: 0.986 ± 0.168 for NNP; HLJ: 0.944 ± 0.497 vs. NM: 0.983 ± 0.650 for BN). PNP showed a striking contrast with NM demonstrating substantially lower tolerance (0.779 ± 0.349) compared to HLJ (0.988 ± 0.335), while SYP revealed NM superiority (1.122 ± 0.545) over HLJ (0.909 ± 0.421), indicating location-specific adaptation strategies for reproductive trait maintenance under shade stress. Additionally, the findings of radar chart analysis (Fig. 4c) revealed distinct tolerance profiles between locations. NM accessions consistently demonstrated superior tolerance indices across most traits, forming a larger polygon area indicating broader adaptive capacity, while HLJ accessions showed a more constrained profile with consistently lower tolerance indices, particularly for reproductive traits. The most pronounced differences occurred in PNP tolerance, where NM accessions showed substantially higher tolerance indices compared to HLJ, indicating superior reproductive resilience under shade stress.

The correlation analysis revealed interdependence among tolerance traits across the six measured parameters (Fig. 4d). For example, NNP tolerance exhibited the strongest correlations, showing moderate relationships with PNP tolerance (*r* = 0.35) and weaker associations with PH tolerance (*r* = 0.25) and BPH tolerance (*r* = 0.14). Also, PNP tolerance demonstrated moderate correlations with BN tolerance (*r* = 0.20) and SYP tolerance (*r* = 0.12), indicating coordinated reproductive responses under shade stress. PH tolerance showed relatively weak correlations with most traits, with the strongest relationship observed with NNP tolerance (*r* = 0.25) and SYP tolerance (*r* = 0.17). BN tolerance showed positive correlations with PNP (*r* = 0.20) and SYP tolerance (*r* = 0.10), while SYP tolerance demonstrated weak to moderate positive correlations across most traits. Notably, negative correlations were observed between BPH and BN tolerance (*r* = −0.15) and between BPH and PNP tolerance (*r* = −0.12).

Shade tolerance classification was conducted using a tolerance index-based approach with four quantitatively defined categories: sensitive (≤ 0.681), moderate (0.681–0.850), tolerant (0.850-1.000), and enhanced (> 1.000). These thresholds were established through statistical analysis of the distribution data and validated against physiological performance metrics. Distribution analysis revealed distinct geographic patterns in shade tolerance levels across the 460 soybean accessions (Fig. 4e, Table**S3**). The classification system successfully differentiated genotypes based on their adaptive responses to shade stress, with geographic distribution showing non-random patterns that suggest environmental selection pressures and adaptive evolution in different regions. At HLJ Location the findings demonstrated a predominance of enhanced tolerance genotypes (222 accessions), followed by tolerant types (169 accessions). Moderate tolerance genotypes were represented by approximately 65 accessions. This distribution indicates that HLJ populations possess exceptional genetic diversity with a strong bias toward superior shade tolerance capacity. At NM, the findings exhibited a markedly different distribution pattern, with enhanced genotypes comprising the largest category (234 accessions), followed by tolerant types (142 accessions). Moderate tolerance accessions numbered approximately 55, while sensitive genotypes were present in small numbers (7 accessions). Despite showing high overall tolerance, NM demonstrated a slightly more pronounced concentration in the enhanced category compared to HLJ. The distribution patterns between locations reveal that both populations are dominated by high-performing genotypes (tolerant and enhanced categories), suggesting strong selective pressure for shade tolerance traits in both geographic regions, with NM showing a slight advantage in enhanced tolerance representation.

To determine the magnitude and real-world significance of shade-induced changes beyond mere statistical differences, Cohen’s d effect size analysis was applied to the six measured traits (Fig. 4f). The largest negative effect size (−0.34) was exhibited by PNP, indicating substantial reduction in reproductive output under shade conditions, where SYP showed the second largest negative effect (−0.30), demonstrating severe impacts on seed production. Also, BN displayed a moderate negative effect (−0.24), while NNP showed the smallest negative impact (−0.10). In contrast, morphological traits showed positive responses to shade treatment such as PH demonstrated a moderate positive effect size (0.33), while BPH exhibited a similar positive effect (0.31). These positive effect sizes confirm the shade avoidance elongation response, where plants increase vertical growth to compete for light resources under shade stress conditions. The results reveal a clear pattern where reproductive traits (SYP, PNP, BN, NNP) were negatively impacted by shade treatment, while morphological traits (PH, BPH) showed adaptive increases, reflecting the trade-off between growth and reproduction under light-limited conditions.

The correlation analysis between overall tolerance index and yield stability index (Fig. 4g) revealed a significant positive relationship (R² = 0.300, *p* < 0.001). Geographic clustering patterns showed distinct separation, with accessions distributed across tolerance ranges from 0.4 to 0.8, and yield stability indices ranging from 0.2 to 1.0. The color gradient indicates location-specific clustering, with genotypes from different geographic origins showing varied tolerance-stability combinations. The positive correlation indicates that genotypes with superior overall stress tolerance consistently demonstrate better yield maintenance under shade conditions, providing a foundation for indirect selection strategies in breeding programs.

The PCA biplot (Fig. 4h) revealed a clear population structure with PC1 (27.0% variance) effectively separating NM and HLJ accessions along the horizontal axis. NM genotypes clustered toward positive PC1 values while HLJ accessions occupied negative PC1 space, confirming that geographic origin represents the primary determinant of tolerance patterns. Notably, the PC2 (17.1% variance) captured within-population variation along the vertical axis, with both groups showing substantial internal diversity ranging from approximately − 2 to + 3 on the PC2 scale. The elliptical clustering patterns indicated clear location-based differentiation while maintaining genetic diversity within each population, with some overlap in the central region suggesting shared tolerance mechanisms between locations.

Furthermore, the identification of elite and shade-adaptive cultivars (Fig. 4i) contextualized performance dynamics under shade stress. Elite stable cultivars were defined as those consistently ranking in the top quartile (Q4) under both control and shade conditions. A total of 120 cultivars (13.7%) fell into this category, demonstrating robust performance stability and high yield potential across environments. In contrast, shade-adaptive cultivars were those that significantly improved their yield ranking under shade, transitioning from lower quartiles under control to Q4 under stress. This group included 29 cultivars (3.3%) originating from Q1, 27 cultivars (3.1%) from Q2, and 44 cultivars (5%) from Q3—totaling 100 cultivars (11.4%) identified as shade-adaptive. These genotypes exhibited marked plasticity, suggesting a strong potential for targeted breeding under intercropping or low-light conditions. Together, elite stable and shade-adaptive cultivars represent valuable genetic resources for enhancing soybean productivity in suboptimal light environments.

Finaly, an analysis of three representative cultivars (Fig. 4j-l) revealed distinct response strategies to shade treatment. The accession YJ252 demonstrated a highly shade-tolerant phenotype with remarkable stability across most traits. Four traits - SYP, NNP, PNP, and BPH - showed virtually identical values between control and shade conditions, as evidenced by overlapping circles. Only BN and PH showed modest responses, with BN decreasing moderately and PH increasing slightly under shade. This constitutive tolerance strategy preserves agricultural performance across varying light environments, making it valuable for intercropping systems. For the accession YJ470 demonstrated selective shade tolerance with SYP, PNP, BN, and BPH maintaining identical values between control and shade conditions, preserving both reproductive output and branching architecture. However, PH and NNP showed clear responses, with PH increasing (typical shade avoidance) and NNP decreasing slightly. The accession YJ204 exhibited a different pattern with SYP, PH, and BN showing identical values between treatments. This cultivar maintained stable yield and plant height simultaneously, suggesting efficient resource allocation. The responsive traits PNP, BPH, and NNP showed clear differences, with PNP decreasing under shade while BPH and NNP showed moderate adjustments. These distinct profiles demonstrate unique shade tolerance strategies: YJ252 exhibits comprehensive stability, YJ470 prioritizes reproductive and branching stability with morphological adjustment, while YJ204 maintains yield and structural height while allowing reproductive component flexibility.

### Machine Learning-Based dissection of shade tolerance patterns

**Fig. 5:**
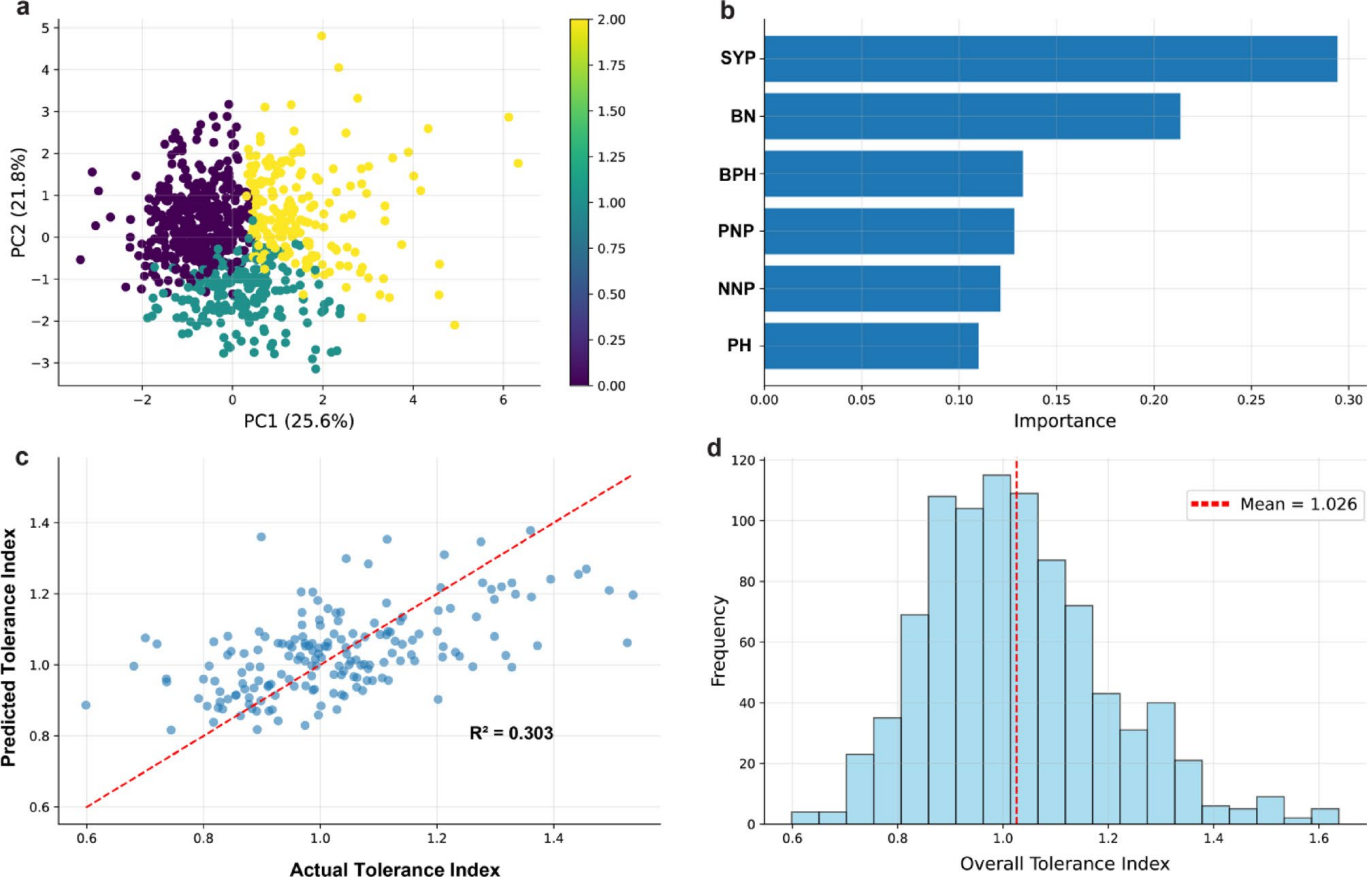
Machine learning-based analysis of overall shade tolerance in soybean accessions. (**a**) K-means clustering of accessions based on principal components of trait-specific tolerance indices, revealing three distinct phenotypic groups. (**b**) Feature importance plot from a random forest model predicting overall tolerance index, highlighting SYP, BN, and BPH as key contributors. (**c**) Scatter plot of predicted vs. actual overall tolerance index values (R² = 0.303), indicating moderate predictive performance of the model. (**d**) Histogram showing the distribution of overall tolerance index values across the population, with a mean of 1.026 (indicated by the red dashed line)

To further investigate the patterns underlying shade tolerance variation in soybean, unsupervised and supervised machine learning approaches were employed. The K-means clustering analysis (Fig. 5a) grouped accessions into three distinct clusters based on principal components derived from trait-specific tolerance indices. The clusters showed clear separation along PC1 (25.6% variance explained), which captured the primary phenotypic gradients associated with shade tolerance, while PC2 (21.8% variance explained) represented secondary axes of trait variation. The color gradient from purple (low tolerance) through teal to yellow (high tolerance) demonstrated a continuous spectrum of shade tolerance responses, with clusters reflecting biologically meaningful differentiation within the shade tolerance landscape. Analyzing analyzed random forest regression model (Fig. 5b) revealed a hierarchical structure in trait contributions to overall shade tolerance prediction. Notably, SYP emerged as the most informative predictor with an important value of approximately 0.30, substantially higher than other traits. This was followed by BN (21.8) and BPH (0.13), showing moderate contribution to as important predictors. The remaining traits, including PNP, NNP, and PH showed progressively lower predictive values, with PH demonstrating the minimal contribution (importance ≈ 0.11), consistent with its previously noted independence from other tolerance components.

The random forest model achieved moderate prediction accuracy with a coefficient of determination (R² = 0.303) between predicted and actual overall tolerance index values (Fig. 5c). The scatter plot revealed a positive correlation with reasonable model performance, though substantial residual variation remained unexplained, suggesting additional factors beyond the measured traits contribute to shade tolerance. The red dashed regression line indicated the model’s capacity to capture major tolerance trends while highlighting the complexity of the underlying biological mechanisms. The distribution of overall tolerance index values across the entire soybean panel (Fig. 5d) followed an approximately normal distribution with a mean of 1.026 (indicated by the red dashed vertical line). The histogram revealed a well-balanced representation of tolerance responses, ranging from highly sensitive genotypes (tolerance index < 0.8) to highly tolerant ones (tolerance index > 1.4). This near-normal distribution pattern indicates that both shade-sensitive and shade-tolerant genotypes are adequately represented in the germplasm collection, providing a valuable genetic resource for breeding programs targeting shade-resilient soybean ideotypes. The frequency distribution peaked around the mean, with gradual tails extending toward both extremes, suggesting quantitative inheritance patterns typical of complex traits influenced by multiple genetic factors.

## Discussion

This study explored the large-scale evaluation of soybean germplasm for shade tolerance has emerged as a critical research priority driven by the increasing adoption of intercropping systems and the need for climate-resilient agricultural practices. Previous studies have employed various approaches to assess soybean responses to light limitation, ranging from small-scale controlled experiments to comprehensive field-based evaluations of hundreds of accessions, revealing substantial genetic variation in morphological, physiological, and yield-related responses to shade stress [8, 19, 28]. This study revealed that shade stress elicits a dualistic response in soybean, characterized by enhanced vertical growth (shade avoidance) alongside a marked reduction in reproductive output. The increase in PH and BPH under shade across both locations aligns with classical shade avoidance syndrome, where plants elongate to outcompete neighbors for light [3, 17, 29–35]. Furthermore, Su et al. [36] demonstrated that soybean plants display a suite of shade-avoidance responses when co-grown with maize; these responses result in a lower photosynthetic capacity, an elongated stem, reduced branching, a higher lodging rate, and lower yield, which directly supports our findings of increased PH height and BPH coupled with reduced reproductive output. These morphological adjustments, while adaptive for light capture, come at a substantial cost to reproductive output, as evidenced by the significant reductions in PNP, BN, and SYP observed in our study. This trade-off is consistent with previous reports on soybean and other crops, where shade reduces reproductive success despite increased stem elongation [8, 37].

This morphological plasticity demonstrates the remarkable adaptive capacity of soybean germplasm. The differential responses between HLJ and NM locations suggest that environmental factors beyond light availability, including temperature regimes, photoperiod sensitivity, and soil characteristics, modulate shade tolerance mechanisms. Zhou et al. [38] reported that the shading of maize is an important factor, which leads to lodging and yield loss of soybean in the maize–soybean strip intercropping system, highlighting the practical importance of understanding these location-specific responses for developing regionally adapted varieties. Noteworthy, the significant location × treatment interactions for reproductive traits (NNP, PNP, SYP) highlight the environmental specificity of shade responses, with HLJ and NM genotypes exhibiting distinct adaptation strategies. Particularly, HLJ cultivars generally showed higher trait values and greater variation, possibly reflecting their adaptation to longer photoperiods and cooler climates, while NM genotypes exhibited stronger shade tolerance indices and a broader tolerance profile. This geographic differentiation aligns with the concept of local adaptation, where soybean populations evolve specific trait combinations suited to their native environments [39]. Such a geographic structure may also suggest that regional selection pressures have shaped divergent adaptive strategies, consistent with patterns observed in other evaluations of stress tolerance. The superior performance of NM accessions was also observed in several tolerance indices, particularly for reproductive traits, which may reflect adaptation to environments with more variable light conditions, resulting in enhanced phenotypic plasticity. The findings of PCA and radar plot analyses for NM accessions indicate superior adaptive capacity across multiple traits, suggesting that this population has evolved more comprehensive stress tolerance mechanisms. This finding has important implications for breeding programs, as it identifies NM germplasm as a valuable source of genes for broad-spectrum shade tolerance.

The positive correlations between trait values under control and shade indicate some genetic stability, but differing correlation strengths show trait plasticity. The strong correlation for PH under both conditions highlights its stability and suggests stem elongation is a conserved response to low light. This consistency makes PH a reliable trait for breeding shade-resistant plants [32, 40–43]. In contrast, the lower correlations for BN and BPH suggest these architectural traits are more environmentally sensitive and thus potential targets for breeding programs aiming to enhance shade tolerance through plasticity [34, 35, 44, 45]. In this study, our path analysis revealed a striking reorganization of trait contributions to seed yield under shade conditions, with NNP emerging as the most critical determinant of reproductive success under light limitation. This shift from PH dominance under control conditions to NNP primacy under shade stress reflects a fundamental change in physiological priorities. Gong et al. [46] found that compared with leaves under control treatment, leaves under shading treatment exhibited decreased palisade and spongy tissue thicknesses, indicating that anatomical modifications accompany the architectural changes we observed, supporting the concept that shade tolerance involves comprehensive physiological reorganization. Moreover, the capacity to maintain node production under reduced light availability becomes critical for preserving reproductive sites and ultimately seed yield, as shade-tolerant genotypes can better regulate their canopy structure to optimize light capture and photosynthesis. Conversely, the intensified negative effect of BPH on yield under shade conditions suggests that excessive stem elongation, while potentially beneficial in reducing light competition, becomes detrimental to overall productivity. This finding supports the concept that optimal shade tolerance requires balanced morphological responses rather than maximum elongation [32, 47].

The positive correlation between the overall tolerance index and yield stability index in this study offers key insights into the mechanisms behind productivity maintenance under shade stress. Wu et al. [48] demonstrated that during shady periods, stem length and stem mass fraction negatively correlated with yield, while stem diameter positively correlated, supporting our findings that specific morphological traits relate differently to yield under shade stress. Classifying accessions into tolerance groups and identifying elite, stable, and shade-adaptive cultivars aids breeding. The dominance of tolerant genotypes in both locations suggests that selection has enhanced shade resilience, which is vital for intercropping and dense planting where light competition is high. Elite cultivars with high yield stability across environments are valuable for broad adaptation, while shade-adaptive cultivars with plasticity enable targeted improvement [30, 31, 33, 49, 50]. Over 25% of accessions showing consistent or adaptable performance under shade are a significant genetic resource for intercropping varieties. Yan et al. [51] emphasized the importance of shade tolerance for soybean productivity in maize fields. Identifying constitutive versus plastic tolerance strategies (YJ252, YJ470, and YJ204) reveals diverse adaptive mechanisms. Constitutive tolerance maintains stable performance across various traits, offering a homeostatic approach, while plastic tolerance adjusts traits to optimize resource allocation under stress. Machine learning shows SYP as the top predictor of shade tolerance, supporting yield-based breeding and recognizing multiple physiological mechanisms. The framework enables the scalable evaluation of germplasm collections to identify promising materials. Rahaman et al. [52] highlighted the potential of high-throughput phenotyping with advanced analytics for assessing morphological traits linked to growth, while Shakoor et al. [53] reviewed technologies that accelerate crop improvement through imaging and data analysis.

## Conclusion

This thorough assessment of 460 soybean accessions across two contrasting geographic regions has offered unprecedented insights into the genetic structure and adaptive mechanisms underlying shade tolerance in soybean germplasm. The study reveals that soybean shade tolerance is a complex trait influenced by location-specific adaptation strategies, with distinct geographic patterns emerging between HLJ and Inner NM populations. Notably, NM accessions displayed a broader adaptive capacity and higher tolerance indices across multiple traits. The identification of 120 elite stable cultivars (13.7%) that maintain consistent high performance across environments, along with 100 shade-adaptive cultivars (11.4%) showing increased plasticity under stress conditions, provides valuable genetic resources for breeding programs focused on intercropping systems and variable light environments. The study also revealed a notable reorganization of trait contributions to yield under shade conditions, with the NNP emerging as the most important factor for reproductive success under light limitation, replacing plant height as the primary yield determinant observed under control conditions.

## Supplementary Information

Below is the link to the electronic supplementary material.

Supplementary Material 1

Supplementary Material 2

Supplementary Material 3

## Data Availability

The data used or analyzed in this manuscript are included in the manuscript, and further inquiries can be directed to the corresponding author.
